# Immunotherapy plus chemotherapy showed superior clinical benefit to chemotherapy alone in advanced NSCLC patients after progression on osimertinib

**DOI:** 10.1111/1759-7714.14271

**Published:** 2021-12-27

**Authors:** Yaping Long, Qi Xiong, Qi Song, Yao Li, Xiaoyan Li, Boyu Qin, Ziwei Huang, Yi Hu, Bo Yang

**Affiliations:** ^1^ School of Medicine Nankai University Tianjin China; ^2^ Department of Oncology Chinese PLA General Hospital Beijing China; ^3^ Department of Graduate Administration Chinese PLA General Hospital Beijing China

**Keywords:** chemotherapy, *EGFR* mutation, immunotherapy, non‐small cell lung cancer, osimertinib

## Abstract

**Background:**

Osimertinib is the standard first‐line treatment for non‐small cell lung cancer (NSCLC) patients with epidermal growth factor receptor (*EGFR*) mutation. Resistance to osimertinib remains a clinical challenge. However, the optimal therapy for these patients is still controversial. In this study, we aimed to assess the efficacy and safety of immunotherapy plus chemotherapy (IO+C) compared with chemotherapy (C) in NSCLC patients after progression on osimertinib.

**Methods:**

Advanced NSCLC patients after progression on osimertinib were retrospectively reviewed. Progression‐free survival (PFS), overall survival (OS), objective response rate (ORR), disease control rate (DCR), and safety were evaluated between the patients treated with IO+C and C.

**Results:**

A total of 40 patients were included in the study. There were 20 patients each in the IO+C group or C group. The ORR was significantly higher in patients in the IO+C group (45% vs. 25%, *p* < 0.01). The median PFS was 6.4 months for patients in the IO+C group compared to 2.8 months for patients in C group (HR: 0.41, 95% confidence interval [CI]: 0.20–0.82, *p* < 0.01). The median OS was significantly longer in the IO+C group than the C group (OS: 12.8 vs. 10.5 months, HR: 0.39, 95% CI: 0.19–0.80, *p* < 0.01). In subgroup analysis, patients of both sexes, age ≤ 65, bone or adrenal metastasis, exon19 del mutation, and third‐line treatment obtained more OS benefits from immunotherapy. The safety profile of both groups was comparable.

**Conclusions:**

Our study provides the clinical evidence of favoring immunotherapy plus chemotherapy in NSCLC patients after progression on osimertinib.

## Graphical abstract

Our study provides the clinical evidence that immunotherapy plus chemotherapy is of more benefit to NSCLC patients after progression on osimertinib in both progression‐free time and overall survival.

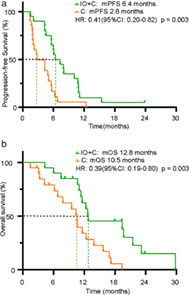

## INTRODUCTION

Non‐small cell lung cancer (NSCLC) is the most common type of cancer with high mortality worldwide. Epidermal growth factor receptor (EGFR)‐tyrosine kinase inhibitors (TKIs) are important milestones in the development of tumor‐targeted therapy and have greatly improved outcomes for NSCLC patients. Osimertinib, the first third‐generation EGFR‐TKI, was initially approved for NSCLC patients with *EGFR* T790M mutations after resistance to first‐generation EGFR‐TKIs.^1^, ^2^ The phase III FLAURA study revealed that NSCLC patients treated with osimertinib had a significantly prolonged progression‐free survival (PFS) and overall survival (OS) than those treated with first‐generation EGFR‐TKI as first‐line treatment (PFS: 18.9 months vs. 10.2 months; OS: 38.6 months vs. 31.8 months).^3^, ^4^ Based on these findings, osimertinib was also approved as the standard first‐line treatment for all *EGFR*‐mutation patients. Unfortunately, patients treated with osimertinib inevitably develop resistance.

Multiple mechanisms are involved in resistance to osimertinib, including *EGFR*‐independent mechanisms and *EGFR*‐dependent mechanisms,^5^ such as tertiary *EGFR* C797S mutation, bypass (c‐MET, HER2) or downstream activation (*RAS* family mutation and amplification), and histological transformation (small‐cell lung cancer transformation and epithelial‐mesenchymal transition).^6^ The resistance mechanism for approximately 50% of patients is unknown.^7^ Thus, the precise mechanism of resistance to osimertinib is not yet fully understood. These make the optimal treatment strategy still controversial.

Immunotherapy targeting the programmed cell death protein 1(PD‐1)/ligand 1 (PD‐L1) pathway has transformed the standard treatment for NSCLC. Multiple studies have shown superior survival benefits for anti‐PD‐1/PD‐L1 inhibitors plus chemotherapy compared to chemotherapy in NSCLC patients without *EGFR* or *ALK* alterations.^8^, ^9^, ^10^, ^11^ In addition, the study IMpower150 has reported the clinical benefit of anti‐PD‐L1 inhibitors in patients with *EGFR* mutations,^12^ while no data is available for patients progressing on osimertinib. White et al. found that immunotherapy plus chemotherapy did not promote PFS or OS in NSCLC patients resistant to osimertinib.^13^ Considering the inconsistent results among studies, the role of immunotherapy in patients with oncogenic drivers is still controversial. Here, we aimed to assess the efficacy and safety of immunotherapy plus chemotherapy (IO+C) compared with chemotherapy (C) in *EGFR*‐mutant NSCLC patients progressing on osimertinib.

## METHODS

### Patients and assessment

Advanced NSCLC patients who experienced progression on osimertinib in the Chinese PLA General Hospital between June 2015 and September 2020 were included. This study was approved by the institutional ethics committee and was conducted by the Declaration of Helsinki. The informed consent was waived due to the retrospective nature of the study.

The inclusion criteria were as follows: (1) Histologically‐ or cytologically‐confirmed lung adenocarcinoma, (2) stage IIIB–IV (American Joint Committee on Cancer eighth edition), (3) documentation of *EGFR* mutation and previously receiving osimertinib, (4) systemic progression on osimertinib with at least one measurable lesion, and (5) treated with anti‐PD‐1/PD‐L1 inhibitors plus chemotherapy or chemotherapy alone after progression on osimertinib. Exclusion criteria were: (1) continuous treatment with osimertinib after progression on osimertinib, (2) Eastern Cooperative Oncology Group Performance Status (ECOG PS) worse than 3, (3) uncontrolled infection, and (4) treatment with immunotherapy before osimertinib.

Demographic data and clinical characteristics, including age, gender, smoking status, baseline *EGFR* mutation status, previous EGFR‐TKI treatment, ECOG PS, radiological and laboratory data were collected from electronic medical records. Follow‐up data were collected up to May 31, 2021. The median follow‐up time was 11.2 months (range: 1.5–29.8 months).

The antitumor response was assessed by Response Evaluation Criteria in Solid Tumors (RECIST) version 1.1. PFS was defined as the duration time from therapy initiation to disease progression or death of any cause before disease progression; OS was defined as the time from the beginning of therapy to death. The rate of complete response (CR) and partial response (PR) was used to calculate ORR, and the rate of CR, PR, and stable disease (SD) was used to calculate DCR. Treatment‐related adverse events (TRAEs) were graded using Common Terminology Criteria for Adverse Events (CTCAE) version 5.0.

### Treatment

The dosages of immunotherapy and chemotherapy for both groups were set according to the National Comprehensive Cancer Network or Chinese Society of Clinical Oncology guidelines. Specifically, the dosages of chemotherapy were 135–175 mg/m2 for paclitaxel, 75 mg/m2 for cis‐platinum, AUC 5–6 for carboplatin, 500 mg/m2 for pemetrexed, and 75 mg/m2 for docetaxel on day 1 every 3 weeks. The dosage for etoposide was 100 mg/m2 on days 1 to 5 every 3 weeks. The dosages of immunotherapy were 240 mg or 3 mg/kg for nivolumab every 2 weeks, 200 mg or 2 mg/kg for pembrolizumab every 3 weeks. The dosage of bevacizumab was 7.5 mg/kg every 3 weeks.

### Statistical analysis

Categorical variables were analyzed using the chi‐square or Fisher's exact test, and continuous variables were analyzed by Mann–Whitney U test or Student's *t*‐test. Analysis of variance (ANOVA) was used to compare the difference among three or more groups. The Kaplan–Meier method was used to estimate OS and PFS. The univariate analysis between groups was assessed by a log‐rank test. Cox regression was used to analyze the statistically significant factors according to the results of univariate analysis. All reported *p*‐values were 2‐tailed, and the difference was considered statistically significant at *p* < 0.05. Statistical analyses were conducted using IBM‐SPSS Statistics version 20 (IBM Corp.). Univariate analysis and multivariate Cox regression hazard analysis were performed by R version 4.1.0.

## RESULTS

### Risk factors related to survival and baseline clinical characteristics in all patients

**FIGURE 1:**
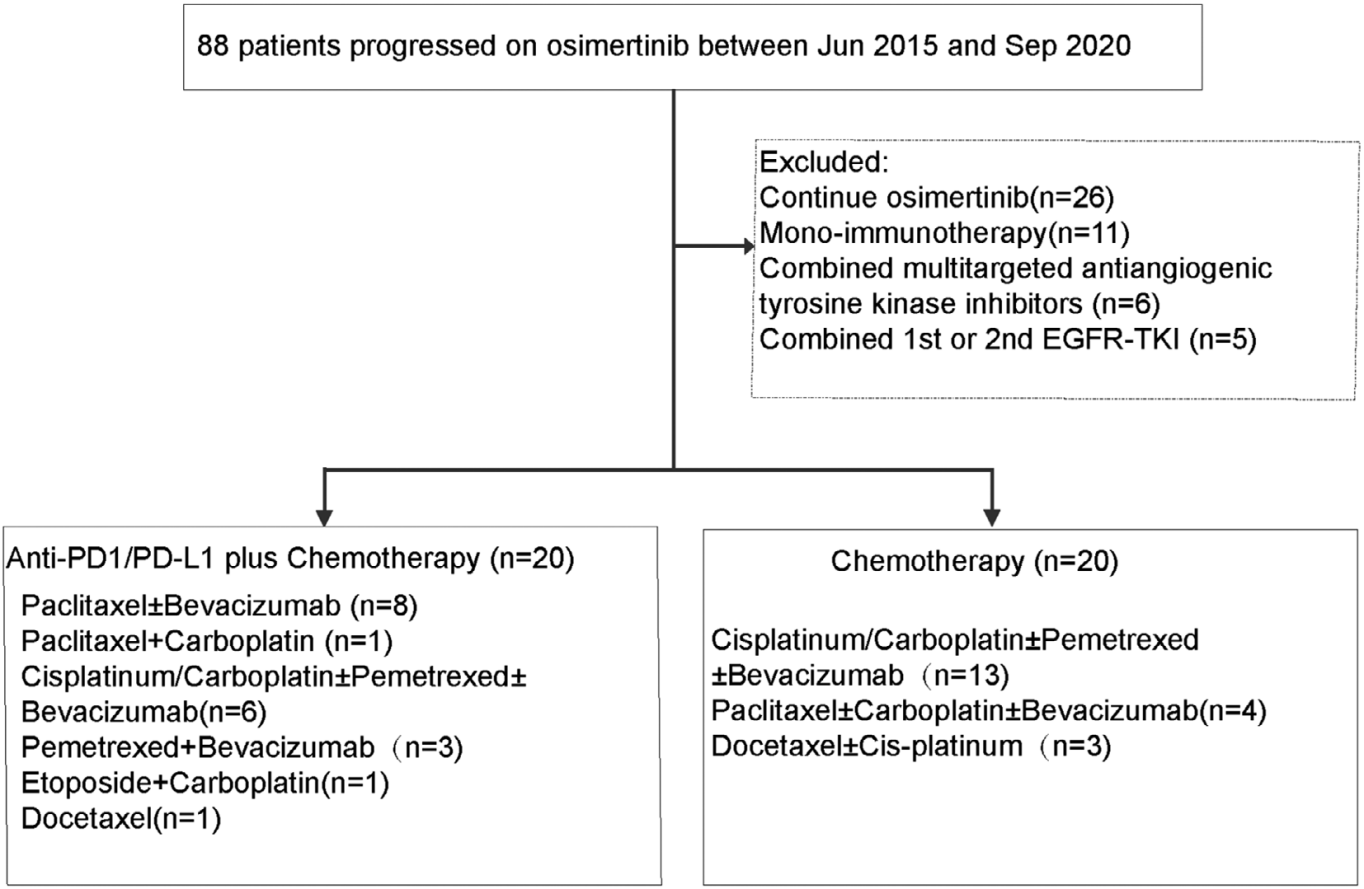
Workflow of the present study

Eighty‐eight advanced NSCLC patients progressing on osimertinib were reviewed, and 40 patients met the inclusion criteria. The univariate analysis indicated that the therapeutic regimen was the only risk factor relevant with OS (12.8 vs. 10.5 months, HR: 0.39, 95% CI: 0.19–0.80, *p* < 0.01) (Supporting Information, Table S1). Hence, the 40 eligible patients were grouped according to the therapeutic regimen. Of the 40 patients, 20 were in the IO+C group, and the other 20 were in the C group. Figure 1 shows the workflow of this study.

All baseline characteristics between the two groups were well balanced. The majority of patients were female (60% vs. 65%), non‐smokers (75% vs. 85%), and ECOG 0–1 (75% vs. 70%) in the IO+C group and C group. A total of 90% of patients were stage IV in both groups, and most patients suffered multiple metastases with bone metastasis being the most frequent. All patients in both groups received two or more lines of previous treatment. With regard to *EGFR* mutation before osimertinib treatment, 10 patients harbored exon 21 L858R mutation, nine patients harbored exon 19 del mutation, and six patients harbored exon 20 T790M mutation in the IO+C group, whereas in the C group, 11 patients had exon 21 L858R mutation, eight patients had exon19 del mutation, and seven patients had exon 20 T790M mutation. Detailed comparisons are shown in Table 1.

**TABLE 1:** Characteristics of patients included in the IO+C and C groups

Subgroup	IO + C (*n* = 20)	C (*n* = 20)	*p*‐value
**Gender, n (%)**			1.0
Female	12 (60)	13 (65)	
Male	8 (40)	7 (35)	
**Age, median (range)**	55 (40–84)	58.5 (44–87)	0.3
**Age, n (%)**			0.7
<=65	17 (85)	15 (75)	
>65	3 (15)	5 (25)	
**Smoker, n (%)**			0.7
No	15 (75)	17 (85)	
Yes	5 (25)	3 (15)	
**ECOG PS, n (%)**			1.0
0–1	15 (75)	14 (70)	
2–3	5 (25)	6 (30)	
**Pathological stage, n (%)**			0.5
Stage IIIB	2 (10)	2 (10)	
Stage IV	18 (90)	18 (90)	
***EGFR* mutation, n (%)**			
Exon18 G719X	1 (5)	0	1.0
Exon19 del	9 (45)	8 (40)	1.0
Exon20 T790M	6 (30)	7 (35)	1.0
Exon21 L858R	10 (50)	11 (55)	1.0
**Platinum‐based chemotherapeutic agents, n (%)**			0.2
No	12 (60)	7 (35)	
Yes	8 (40)	13 (65)	
**Metastasis, n (%)**			
Liver metastasis	7 (35)	6 (30)	1.0
Brain metastasis	10 (50)	11 (55)	1.0
Bone metastasis	14 (70)	16 (80)	0.7
Adrenal metastasis	5 (25)	4 (20)	1.0
**Number of treatment lines, n (%)**			0.5
Third	12 (60)	13 (55)	
Beyond fourth	8 (40)	7 (35)	

*Abbreviations*: C, chemotherapy; ECOG PS, Eastern Cooperative Oncology Group Performance Status; *EGFR*, epidermal growth factor receptor; IO+C, immunotherapy plus chemotherapy.

### Clinical response

The clinical response was compared between the two groups. Our results showed that ORR (45% vs. 25%, *p* < 0.01) and DCR (85% vs. 65%, *p* = 0.03) were significantly higher in patients in the IO+C group than those in the C group. In the IO+C group, nine (45%) patients achieved PR, eight (40%) patients gained SD, and three (15%) patients suffered progressive disease (PD). In the C group, the numbers of patients who experienced PR, SD, and PD were five (25%), six (30%), and nine (45%), respectively.

### Comparison of PFS between patients in IO+C group and C group

**FIGURE 2:**
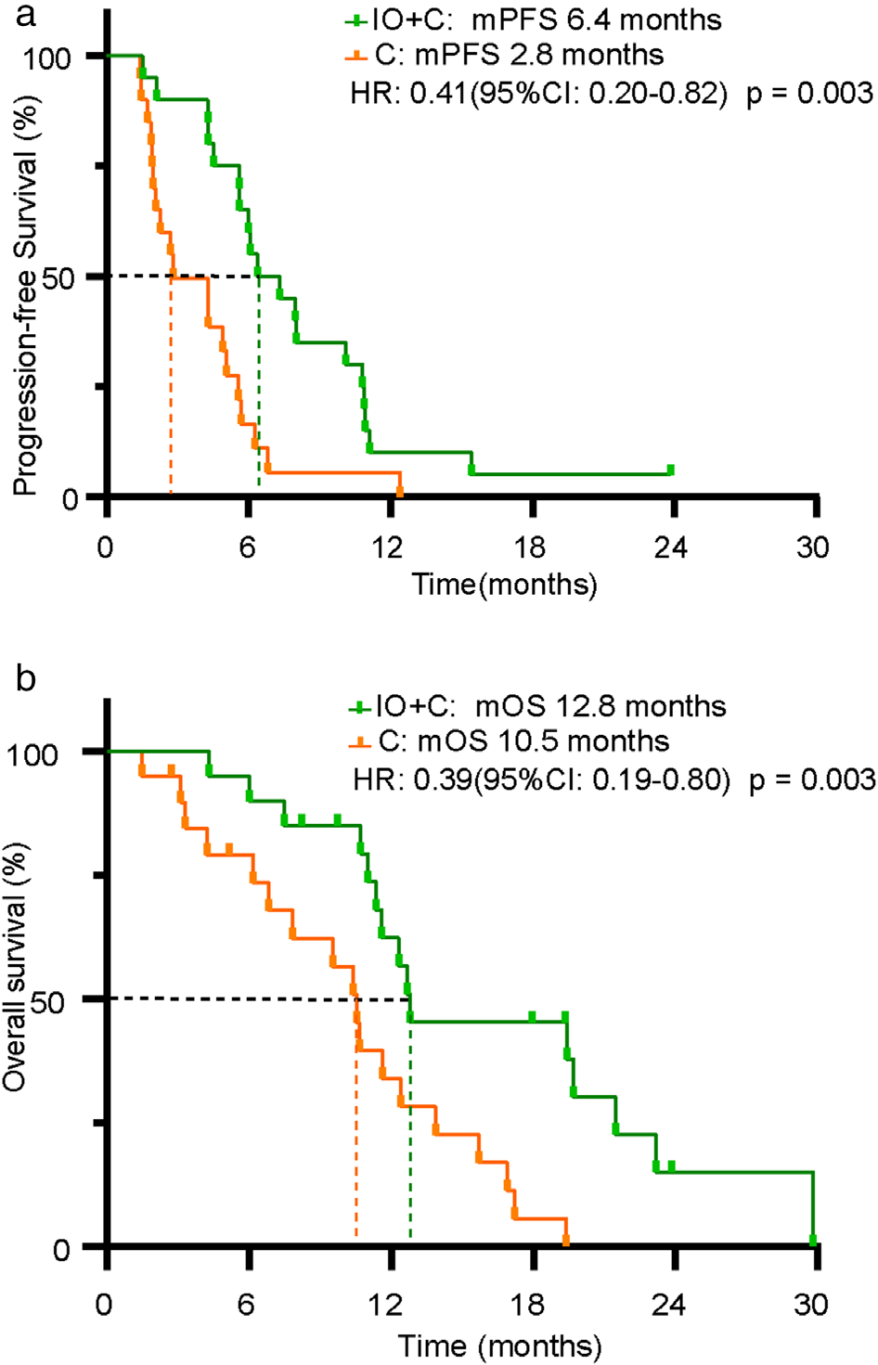
Survival comparison between patients in the IO+C and C groups. (a) Comparison of progression‐free survival (PFS). (b) Comparison of overall survival (OS)

**FIGURE 3:**
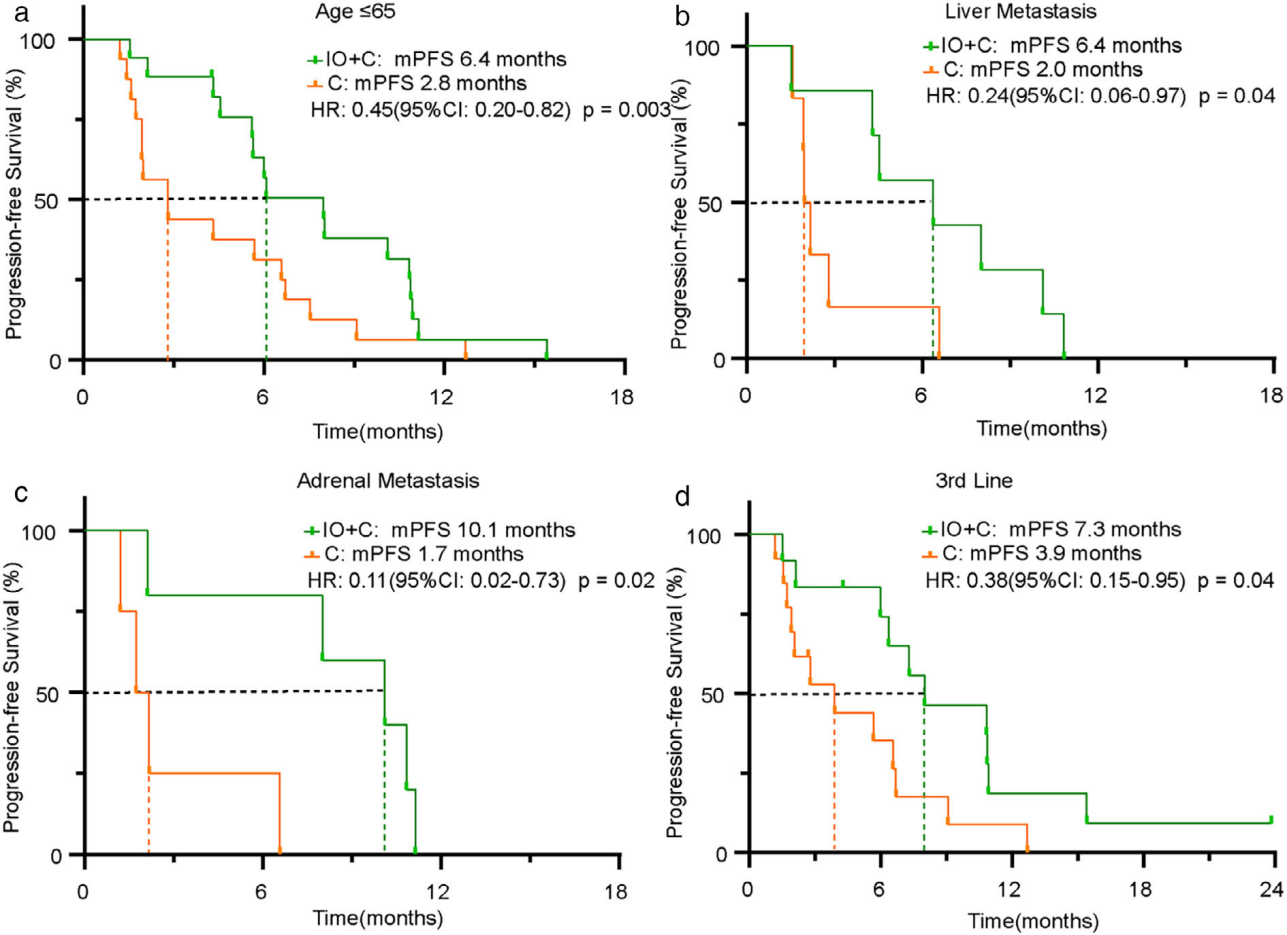
Subgroup analysis of PFS between patients in the IO+C and C groups. (a) Age ≤ 65 subgroup. (b) Liver metastasis subgroup. (c) Adrenal metastasis subgroup. (d) Third‐line treatment subgroup

The median PFS was significantly longer in patients in the IO+C group than those in the C group (6.4 vs. 2.8 months, HR: 0.41, 95% CI: 0.20–0.82, *p* < 0.01) (Figure 2a). To identify the patients who most probably benefit from the addition of immunotherapy, subgroup analysis of PFS was performed. Our results indicated that patients age ≤ 65, liver metastasis, adrenal metastasis, and third‐line treatment had significantly prolonged PFS in the IO+C group compared with those in the C group (Figure 3, Table 2). Notably, the number of patients in the adrenal metastasis and liver metastasis subgroups was relatively small. In addition, our results also showed that patients in subgroups of both sexes, bone or brain metastasis and exon 19 del or exon 21 L858R mutation showed a trend of longer PFS in the IO+C group (Supporting Information, Figure S1).

**TABLE 2:** Subgroup analysis of PFS and OS between patients in IO+C and C groups

Subgroup	PFS (months)							OS (months)				
	IO + C			C				IO + C		C		
	*n*	mPFS	95% CI	*n*	mPFS	95% CI	*p*‐value	mOS	95% CI	mOS	95% CI	*p*‐value
**Gender**							0.1					0.003
Female	13	6.0	5.2–6.8	12	3.9	1.4–6.4		12.8	3.2–22.4	11.6	8.2–15.1	
Male	7	8.0	3.0–12.9	8	2.8	1.1–4.5		12.3	10.5–14.1	10.4	2.5–18.3	
**Age**							0.04					0.003
<65	17	6.1	2.9–9.2	15	2.8	0–5.7		12.7	10.9–14.4	10.5	8.7–12.3	
≥65	3	7.3	5.8–8.8	5	3.9	1.2–6.6		19.4	6.9–32.0	6.2	0–20.0	
**Smoker**							0.09					‐
No	15	6.4	4.7–8.0	17	2.8	0.5–5.1		12.8	10.6–15.0	10.5	8.6–12.4	
Yes	5	8.0	2.9–13.1	3	9.1	NR		23.2	NR	7.8	NR	
**ECOG**							0.04					0.003
0–1	15	6.4	4.3–8.5	14	2.8	0.8–4.8		12.3	10.5–14.1	10.4	4.6–16.2	
2–3	5	8.0	3.9–12.1	6	2.2	0–8.3		21.5	2.9–40.1	2.3	6.0–15.1	
**EGFR mutation**												
Exon19 del mutation	9	8.0	1.0–14.9	8	2.8	1.9–3.7	0.07	19.7	2.5–36.9	10.4	8.2–12.6	
Exon20 T790M mutation	6	5.6	5.2–6.1	7	4.3	3.3–5.3	0.08	11.0	10.3–11.7	10.67	0.8–20.5	
Exon21 L858R mutation	10	6.4	4.5–8.3	11	3.9	0.02–7.8	0.09	11.6	9.6–13.6	10.5	5.0–16.1	
**Liver metastasis**							0.02					0.004
No	13	7.3	5.0–9.6	14	4.3	1.3–7.3		12.7	1.9–23.5	10.7	9.2–12.1	
Yes	7	6.4	1.6–11.1	6	2.0	1.7–2.3		12.8	9.1–16.5	6.8	0–13.7	
**Brain metastasis**							0.2					0.006
No	10	10.1	4.6–15.6	9	3.9	0–8.6		19.4	5.3–33.6	10.5	2.1–18.9	
Yes	10	5.6	5.0–6.3	11	2.8	0.5–5.1		12.7	10.2–15.1	10.7	9.0–12.4	
**Bone metastasis**							0.09					0.006
No	6	8.0	0.1–15.9	4	2.0	0.2–3.7		29.8		3.3	0.6–6.0	
Yes	14	6.1	5.4–6.7	16	2.8	0.02–5.6		12.7	11.0–14.3	10.7	9.1–12.2	
**Adrenal metastasis**							0.03					0.002
No	15	6.1	5.1–7.0	16	3.9	1.2–6.6		12.8	3.5–22.1	11.6	9.6–13.6	
Yes	5	10.1	5.6–14.6	4	1.7	0.8–2.7		12.3	10.3–14.3	3.1	0.4–5.8	
**Platinum‐based regimens**							0.01					0.009
No	12	6.1	5.4–6.7	7	2.0	1.9–2.1		11.6	9.6–13.6	11.6	9.6–13.7	
Yes	8	7.3	2.5–12.1	13	5.7	1.0–10.3		19.4	8.9–30.0	9.5	5.8–13.3	
**Line of combination therapy**							0.05					0.004
Third	12	7.3	4.5–10.0	13	3.9	1.0–6.8		19.4	3.7–35.2	10.7	8.7–12.6	
Fourth or beyond	8	5.6	5.0–6.3	7	2.8	1.2–4.4		12.3	9.2–15.4	9.5	5.2–13.9	

*Abbreviations*: C, chemotherapy; EGFR, epidermal growth factor receptor; IO+C, immunotherapy plus chemotherapy; OS, overall survival; PFS, progression‐free survival.

### Comparison of OS between patients in IO+C group and C group

**FIGURE 4:**
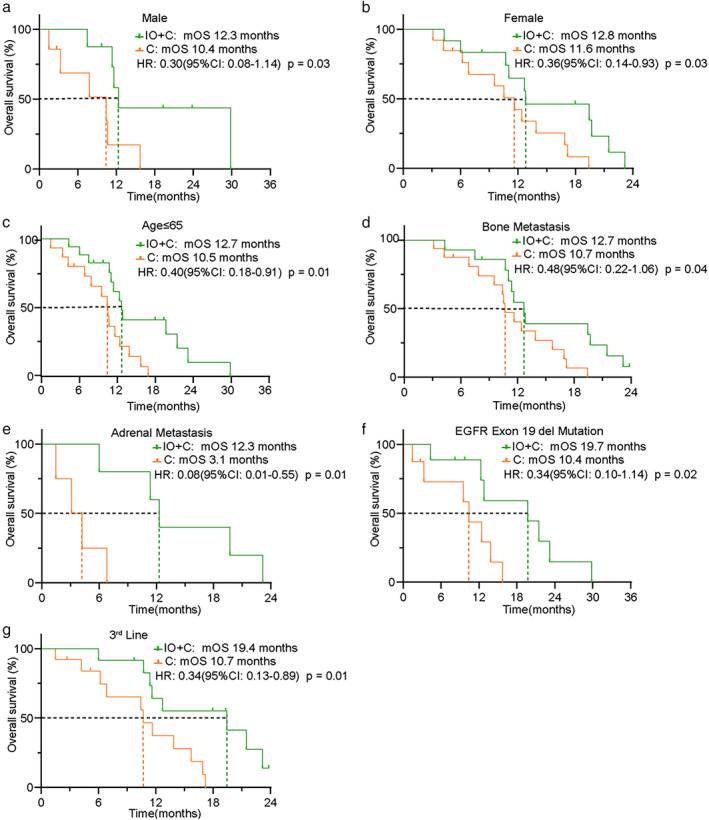
Subgroup analysis of OS between patients in the IO+C and C groups. (a) Male subgroup. (b) Female subgroup. (c) Age ≤ 65 subgroup. (d) Bone metastasis subgroup. (e) Adrenal metastasis subgroup. (f) EGFR exon19 del mutation subgroup. (g) Third‐line treatment subgroup

The median OS was also significantly prolonged in patients in the IO+C group compared to the C group (12.8 vs. 10.5 months; HR 0.39, 95% CI: 0.19–0.80; *p* < 0.01) (Figure 2b). Subgroup analysis was subsequently conducted. Similar to the results of PFS, our results demonstrated that the OS for patients of both sexes, aged ≤65, adrenal metastasis, exon 19 del mutation, and third‐line treatment was longer in the IO+C group than in the C group. Furthermore, patients with bone metastasis had marginally significantly longer OS in the IO+C group than the C group (Figure 4, Table 2). Our results also showed that the OS in subgroups of exon 21 L858R mutation and liver or brain metastasis was longer in the IO+C group, though no significant difference was found (Supporting Information, Figure S2).

### Comparison of safety between patients in IO+C group and C group

TRAEs were reported in 85% of patients in the IO+C group and 90% of patients in the C group. In the IO+C group, two patients experienced grade three or higher anemia and one patient reported grade 3 or higher thrombocytopenia. The most frequent TRAEs in the IO+C group were anemia, increased aspartate transaminase or alanine aminotransferase, hypocalcemia, and leukopenia (with 25% or more incidence). In the C group, grade 3 or higher anemia, leukopenia, and thrombocytopenia were reported in one, two, and two patients, respectively. Similar to patients in the IO+C group, the most frequent TRAEs in the C group were anemia, hypocalcemia, weakness, hypophosphatemia, and thrombocytopenia (with 30% or more incidence). No treatment‐related death or interstitial lung disease was recorded in our study (Table 3).

**TABLE 3:** Incidence of TRAEs between patients in IO+C group and C group

Events	IO + C, n (%)		C, n (%)	
	Grade 3–4	Grade 1–2	Grade 3–4	Grade 1–2
Treatment‐related adverse events	3 (15)	17(85)	3 (15)	18 (90)
Anemia	2 (10)	11 (55)	1 (5)	12 (60)
Decreased neutrophil count	0 (0)	5 (25)	2 (10)	5 (25)
Decreased lymphocyte count	0 (0)	3 (15)	0 (0)	3 (15)
Decreased platelet count	1 (5)	1 (5)	2 (10)	6 (30)
Elevated aspartate transaminase	0 (0)	8 (40)	0 (0)	5 (25)
Elevated alanine transaminase	0 (0)	6 (30)	0 (0)	5 (25)
Elevated creatinine	0 (0)	1 (5)	0 (0)	2 (10)
Hypokalemia	0 (0)	2 (10)	0 (0)	3 (15)
Hypocalcemia	0 (0)	6 (30)	0 (0)	7 (35)
Hypophosphatemia	0 (0)	5 (25)	0 (0)	6 (30)
Thyroid dysfunction	0 (0)	2 (10)	0 (0)	0 (0)
Diarrhea	0 (0)	0 (0)	0 (0)	1 (5)
Anorexia	0 (0)	2 (10)	0 (0)	3 (15)
Nausea	0 (0)	5 (25)	0 (0)	6 (30)
Vomiting	0 (0)	5 (25)	0 (0)	3 (15)
Weakness	0 (0)	5 (25)	0 (0)	7 (35)
Fever	0 (0)	2 (10)	0 (0)	0 (0)
Alopecia	0 (0)	0 (0)	0 (0)	2 (10)
Hemorrhage	0 (0)	0 (0)	0 (0)	2 (10)
Cough	0 (0)	0 (0)	0 (0)	4 (20)
Chest tightness	0 (0)	1 (5)	0 (0)	3 (15)
Panting	0 (0)	1 (5)	0 (0)	1 (5)
Cardiopalmus	0 (0)	2 (10)	0 (0)	1 (5)

*Abbreviations*: C, chemotherapy; IO+C, immunotherapy plus chemotherapy.

## DISCUSSION

Currently, chemotherapy is considered standard therapy for patients after progression on osimertinib. In the present study, we showed that NSCLC patients treated with anti‐PD‐1/PD‐L1 inhibitors combined with chemotherapy achieved better PFS and OS than those treated with chemotherapy alone after progression on osimertinib. Moreover, we found that patients with age ≤ 65, liver or adrenal metastasis, and third‐line treatment gained more PFS benefit when treated with anti‐PD‐1/PD‐L1 inhibitors plus chemotherapy, whereas patients of both sexes, age ≤ 65, bone or adrenal metastasis, exon 19 del mutation and third‐line treatment had significantly prolonged OS with the addition of anti‐PD‐1/PD‐L1 inhibitors. In summary, our results suggested that NSCLC patients after progression on osimertinib could benefit from immunotherapy.

Whether patients with *EGFR* mutation benefit from immunotherapy is still controversial. Previous clinical trials, including CheckMate153, NCT02879994, IMpower130, and WJOG8515L, reported a lack of efficiency in immunotherapy alone in patients with *EGFR* or *ALK* mutation.^9^, ^10^, ^14^, ^15^, ^16^, ^17^, ^18^ Previous studies by Zhang et al. and Lam et al. showed that anti‐PD‐1 inhibitors in combination with chemotherapy achieved promising efficacy in patients with *EGFR* mutation after prior TKI treatment.^19^, ^20^ Notably, there were over 50% of patients who had previously been treated with prior osimertinib.^20^ Furthermore, the study IMpower150 demonstrated that the addition of anti‐PD‐L1 inhibitor to bevacizumab plus chemotherapy, but not chemotherapy alone, improved PFS and OS in patients with *EGFR* sensitizing mutations and treated with one or more TKIs.^12^ During the 2021 European Lung Cancer Virtual Congress, White et al. reported that the addition of immunotherapy to chemotherapy did not show clinical benefit in patients after progression on osimertinib in a retrospective study.^13^ Whereas, our study showed that immunotherapy plus chemotherapy prolonged PFS and OS in patients after progression on osimertinib. We inferred the inconsistency lay in different institution and inclusion criteria, as they had included patients who continued on osimertinib after progression, while those were excluded in our study. In addition, all patients included in our study had received two or more lines of treatment.

Preclinical studies have suggested that patients with high tumor mutational load, high levels of tumor‐infiltrating T cells, and high expression of PD‐L1 would benefit more from anti‐PD‐1/PD‐L1 inhibitors.^21^ Paradoxically, patients with *EGFR* mutations are reported to have lower PD‐L1 expression and tumor mutational burden (TMB), leading to poor response to anti‐PD‐1/PD‐L1 inhibitors.^22^, ^23^ Unlike patients with common *EGFR* mutations, recent studies have found that patients harboring uncommon *EGFR* mutations could benefit more from anti‐PD‐1/PD‐L1 inhibitors.^17^, ^24^ Schmid et al. reported that the mechanism of osimertinib resistance includes *EGFR*‐dependent and *EGFR*‐independent manner. They summarized that most patients resistant to osimertinib harbored uncommon *EGFR* mutations.^25^ Yet, the resistance mechanism for approximately 50% of patient progression on osimertinib is unknown.^25^ Unfortunately, we could not evaluate the gene mutation status of patients after progression on osimertinib in the present study since most patients were treated heavily and tissue biopsy was hard to obtain. Taken together, we speculated that these findings might partially explain the clinical benefit of immunotherapy in patients’ progression on osimertinib in our study, and future studies on the molecular mechanism would help explain the benefit from the addition of immunotherapy to chemotherapy after progression on osimertinib.

In the present study, PFS and OS for patients in subgroups of age ≤ 65, adrenal metastasis, and third‐line treatment were significantly improved. As for the relationship between age and response to anti‐PD‐1 inhibitor, Kugel et al. found that melanoma patients over 60 years old responded better to anti‐PD‐1. They also suggested that the population of regulatory T cells was significantly higher in young mice in a melanoma animal model.^26^ However, similar to our findings, Landre et al. indicated that NSCLC patients older than 75 years had an inferior OS compared with those of a younger age when treated with anti‐PD‐1 inhibitors.^27^ Thus, age is related to the clinical benefit of immunotherapy. The adrenal gland is one of the most frequent metastatic sites in lung cancer. Several case reports of melanoma and colorectal cancer, but not lung cancer, have suggested that adrenal metastasis poorly responded to immunotherapy by decreasing antigen presentation,^28^, ^29^, ^30^ which was contrary to our results. It has been reported that chemotherapy could potentiate anticancer immunity by upregulating the release of immunogenic tumor antigens.^31^ Thus, it is rational to combine immunotherapy with chemotherapy to enhance treatment response. The phase III clinical trials KEYNOTE‐407, KEYNOTE‐189, and IMpower150 have indicated that a combination of chemotherapy and anti‐PD‐1/PD‐L1 inhibitors improved antitumor efficacy than chemotherapy alone in NSCLC patients.^12^, ^32^, ^33^ We speculated the strategy of immunotherapy combined with chemotherapy may contribute to differences.

In terms of safety, most of the adverse events were grades 1 or 2 and the adverse‐event profiles were similar between patients receiving anti‐PD‐1/PD‐L1 inhibitor plus chemotherapy and chemotherapy alone. Most of the adverse events were manageable and no treatment‐related death was observed. No additional safety signal was observed in our study compared to a previous study.^34^ Given the manageable safety profile, the combination of immunotherapy and chemotherapy is well tolerated in NSCLC patients after progression on osimertinib.

Some limitations of our study should be noted. First, this study was a retrospective analysis of data in a single center with a relatively small sample size, which might lead to inevitable bias and weaken the level of evidence. Second, the gene mutation status, PD‐L1 expression, TMB, and microsatellite instability were not accessed; thus, subgroup analysis of these biomarkers could not be performed to identify the patients which benefit most from immunotherapy. Therefore, prospective studies with larger sample sizes and molecular mechanisms are needed to confirm our findings. Indeed, there are some ongoing clinical trials (KEYNOTE‐789, CheckMate722) comparing the efficacy of anti‐PD‐1/PD‐L1 inhibitors plus chemotherapy with chemotherapy alone in NSCLC patients after progression on TKI treatment, including those treated with osimertinib. We are expecting the data of these clinical trials soon.

In conclusion, our study provides clinical evidence of favoring anti‐PD‐1/PD‐L1 inhibitors plus chemotherapy in NSCLC patients after progression on osimertinib. Our study also indicated that patients with age ≤ 65, adrenal metastasis, and third‐line treatment are most likely to benefit from immunotherapy.

## CONFLICT OF INTEREST

The authors have no relevant financial or nonfinancial interests to disclose.

## Supporting information


**Table S1**. Univariate analysis of patients included.
