# Supplementary material for: Immunotherapy plus chemotherapy showed superior clinical benefit to chemotherapy alone in advanced NSCLC patients after progression on osimertinib
Source: Thorac Cancer. 2021 Dec 27;13(3):394–403. doi: 10.1111/1759-7714.14271 (PMC8807266; doi:10.1111/1759-7714.14271)
Supplement: Supplementary file 1 — Table S1. Univariate analysis of patients included. [file TCA-13-394-s001.docx]

Table S1. Univariate analysis of patients included.

|  | **PFS (months)** | | **OS (months)** | |
| --- | --- | --- | --- | --- |
| **Characteristic** | **mPFS (95%CI)** | ***p* value** | **mOS (95%CI)** | ***p* value** |
| **Gender** |  | 0.07 |  | 0.7 |
| Female | 5.6 (3.4-7.8) |  | 12.4 (9.9-14.9) |  |
| Male | 8.0 (3.8-12.2) |  | 11.4 (9.8-12.9) |  |
| **Age, n** |  | 0.2 |  | 0.5 |
| <65 | 5.6 (3.6-7.7) |  | 11.4 (9.3-13.4) |  |
| ≥65 | 6.4 (0.3-12.4) |  | 17.2 (2.8-31.6) |  |
| **Smoker** |  | 0.3 |  | 0.7 |
| No | 5.6 (3.2-8.0) |  | 11.6 (9.8-13.4) |  |
| Yes | 8.0 (2.0-14.0) |  | 12.3 (7.4-17.2) |  |
| **ECOG PS** |  | 0.3 |  | 0.4 |
| 0-1 | 5.6 (4.2-7.1) |  | 11.6 (9.7-13.5) |  |
| 2-3 | 6.7 (3.5-9.9) |  | 12.8 (9.7-15.9) |  |
| **EGFR mutation** |  |  |  |  |
| Exon18 G719X mutation | 8.0 | 0.8 | 29.8 | 0.1 |
| Exon18 wildtype | 5.7 (3.8-7.5) |  | 11.6 (9.8-13.4) |  |
| Exon19 del mutation | 4.5 (4.2-4.9) | 0.6 | 12.8 (11.9-13.7) | 0.5 |
| Exon19 wildtype | 6.1 (5.0-7.2) |  | 11.4 (10.3-12.4) |  |
| Exon20 T790M mutation | 5.6 (3.6-7.6) | 0.5 | 11.0 (10.4-11.6) | 0.2 |
| Exon20 wildtype | 6.4 (3.4-9.3) |  | 12.3 (10.4-14.2) |  |
| Exon21 L858R mutation | 6.1 (5.0-7.1) | 0.5 | 11.6 (10.7-12.5) | 0.7 |
| Exon21 wildtype | 4.5 (3.2-5.9) |  | 12.4 (9.8-15.0) |  |
| **Liver metastasis** |  | 0.07 |  | 0.4 |
| No | 6.1 (4.7-7.4) |  | 12.3 (11.1-13.5) |  |
| Yes | 4.3 (1.5-7.1) |  | 11.4 (8.4-14.4) |  |
| **Brain metastasis** |  | 0.1 |  | 0.5 |
| No | 7.3 (4.8-9.8) |  | 12.3 (9.0-15.6) |  |
| Yes | 5.6 (3.6-7.6) |  | 11.0 (9.3-12.7) |  |
| **Bone metastasis** |  | 0.7 |  | 0.5 |
| No | 4.3 (0-9.9) |  | 12.3 (0-27.1) |  |
| Yes | 5.7 (5.0-6.3) |  | 11.6 (9.9-13.4) |  |
| **Adrenal metastasis** |  | 0.8 |  | 0.2 |
| No | 5.7 (3.7-7.6) |  | 12.4 (10.9-13.9) |  |
| Yes | 6.6 (0-19.4) |  | 6.8 (4.4-9.3) |  |
| **Therapeutic regimen** |  | 0.05 |  | 0.003 |
| IO+C | 6.4 (3.7-9.1) |  | 12.8 (4.1-21.5) |  |
| C | 2.8 (0.4-5.2) |  | 10.5 (9.0-12.1) |  |
| **Platinum based regimens** |  | 0.7 |  | 0.4 |
| No | 6.6 (3.5-9.7) |  | 12.3 (9.7-14.9) |  |
| Yes | 5.6 (3.2-8.1) |  | 11.6 (10.1-13.1) |  |

Abbreviations: PFS, progression-free survival; OS, overall survival; IO+C, immunotherapy plus chemotherapy; C, chemotherapy; ECOG PS, Eastern Cooperative Oncology Group Performance Status; EGFR, epidermal growth factor receptor.
